# Association between Serum Ferritin and Contrast-Induced Nephropathy in Patients with Acute Coronary Syndrome Undergoing Percutaneous Coronary Intervention

**DOI:** 10.1155/2016/5420345

**Published:** 2016-07-28

**Authors:** Boqian Zhu, Jiantong Hou, Yaoyao Gong, Gaoliang Yan, Qingjie Wang, Dong Wang, Yong Qiao, Yifei Chen, Chengchun Tang

**Affiliations:** ^1^Department of Cardiology, Zhongda Hospital of Southeast University Medical School, Nanjing 210009, China; ^2^Department of Gastroenterology, The First Affiliated Hospital of Nanjing Medical University, Nanjing 210029, China; ^3^Department of Cardiology, Xishan People's Hospital, Wuxi Branch of Zhongda Hospital, Wuxi 214000, China

## Abstract

*Background and Aims*. CIN is a major and serious complication following PCI in patients with ACS. It is unclear whether a higher serum ferritin level is associated with an increased risk of CIN in high-risk patients. Thus, we conducted this study to assess the predictive value of SF for the risk of CIN after PCI.* Methods*. We prospectively examined SF levels in 548 patients with ACS before undergoing PCI. Multivariate logistic regression analysis was used to analyze the independent risk factors for CIN. The ROC analysis was performed to evaluate the predictive value of SF for CIN.* Results*. CIN occurred in 96 patients. Baseline SF was higher in patients who developed CIN compared to those who did not (257.05 ± 93.98 versus 211.67 ± 106.65; *P* < 0.001). Multivariate logistic regression analysis showed that SF was an independent predictor of CIN (OR, 1.008; 95% CI, 1.003–1.013; *P* = 0.002). The area under ROC curve for SF was 0.629, and SF > 180.9 *μ*g/L predicted CIN with sensitivity of 80.2% and specificity of 41.4%.* Conclusion*. Our data show that a higher SF level was significantly associated with an increased risk of CIN after PCI.

## 1. Introduction

Contrast-induced nephropathy (CIN) is a relatively infrequent complication of percutaneous coronary intervention (PCI) [1]. However, patients with acute coronary syndrome (ACS) undergoing emergent PCI are at a significantly greater risk, and patients with diabetes or chronic kidney disease (CKD) have a risk of almost 50% [2, 3]. CIN is currently defined either as an acute decrease in renal function after contrast exposure without evidence of other causes or as a rise in serum creatinine (SCr) of 0.5 mg/dL or a 25% increase from the baseline values within 48 to 72 hours following contrast media administration [4]. Development of CIN after PCI is associated with poor clinical outcomes, including late cardiovascular events and high short- and long-term mortality [5]. In clinical practice, some hysteresis is considered to be present in the diagnosis of CIN based on serum creatinine level [4]. Therefore, the search for more rapid, reliable, and sensitive indicators is critical for early diagnosis and screening of patients at high risk of developing CIN.

It is well known that inflammation plays an important role in the pathogenesis of CKD and CIN [5]. Accumulating clinical evidence has demonstrated that inflammatory cytokines such as high-sensitivity C-reactive protein (hs-CRP) and interleukin-18 are independent predictors of CIN after PCI [6–8]. Serum ferritin (SF), a biomarker of iron stores, is also an acute-phase reactant increasing during inflammation. Previous studies have found that SF is closely related to acute renal function decline in CKD patients and pathologic changes of diabetic nephropathy [9–11]. Additionally, SF was also reported to be a strong predictor of morbidity and mortality in hemodialysis and peritoneal dialysis patients [12–14].

However, no relevant report is currently available regarding whether SF is associated with CIN after PCI in ACS patients. In the present study, we aimed to investigate whether SF can be used preoperatively as an early diagnostic marker of CIN onset after PCI in patients with ACS.

## 2. Materials and Methods

### 2.1. Study Subjects

In this prospective observational study, 548 adult patients with ACS between October 2013 and June 2015 who agreed to receive primary PCI treatment in the Cardiology Department of Zhongda Hospital Southeast University were enrolled. The exclusion criteria were as follows: (1) pregnancy; (2) end-stage renal disease requiring dialysis; (3) known allergy to contrast agent; (4) intravascular administration of contrast medium within 7 days; (5) being accompanied with other diseases including autoimmune diseases, hepatic insufficiency, malignant tumor, and hematology disease; (6) oral iron supplement; and (7) cardiac shock. All protocols were approved by the medical ethics committee of the hospital, and all patients provided informed consent.

### 2.2. Study Protocol and Definition

Baseline SF and SCr levels were tested before angiography. Routine analysis of blood, uric acid, low-density lipoprotein cholesterol (LDL-C), hs-CRP, and cystatin C and markers of myocardial injury were also measured. SF was measured using chemiluminescence enzyme immunoassay on UniCel-Dxl 800 Immunoassay System (Beckman Coulter, USA). SCr, uric acid, LDL-C, and cystatin C were all tested on AU5800 autobiochemistry analyzer (Beckman Coulter). hs-CRP was measured by particle enhanced immunonephelometry on BNII system (Siemens Healthcare Diagnostics, Germany). Regular renal function test including SCr during 48–72 hours following PCI was performed to diagnose CIN. CIN was defined as an increase in SCr level of ≥0.5 mg/dL or ≥25% above baseline within 72 hours after PCI. The patients were divided into CIN and non-CIN groups depending on the presence or absence of CIN, respectively. The estimated glomerular filtration rate (eGFR) for each patient was calculated using the Modification of Diet in Renal Disease (MDRD) formula and eGFR < 60 mL/min/1.73 m^2^ was defined in the study as decreased renal function. Left ventricular ejection fraction (LVEF) was measured using echocardiography. The diagnosis of ST-elevation MI (STEMI) and non-ST-elevation MI (NSTEMI) was based on a typical history of chest pain and elevation of cardiac markers, with or without ST segment elevation in electrocardiogram.

### 2.3. Coronary Interventions and Medications

Coronary angiography and PCI were performed by cardiologists specialized in intervention treatment according to standard clinical practice. PCI of diseased vessels was conducted on the basis of coronary angiography findings. The contrast medium applied was iopromide (Bayer HealthCare, USA) or iodixanol (GE healthcare, UK). Both of these agents are nonionic with low osmolarity or isoosmolarity. Hydration therapy was given as determined by the interventional cardiologist depending on the condition of patient. During hydration, isotonic saline solution was intravenously given at 1.0–1.5 mL/kg/h for 3–12 h preoperatively and for 6–24 h postoperatively. After PCI, all patients continued to take aspirin (100 mg/d orally) indefinitely and clopidogrel (75 mg/d) for at least 12 months. Use of other medications (*β*-receptor blockers, angiotensin-converting enzyme inhibitor or angiotensin receptor blocker, nitrate esters, and calcium antagonist) was left to the discretion of individual cardiologist.

### 2.4. Statistical Analysis

Data were analyzed using the Statistical Package for the Social Sciences (SPSS) for Windows, version 13.0. The measured data were expressed as mean ± standard deviation or median and interquartile range. The Kolmogorov-Smirnov (K-S) test was applied to test for normal distribution. Continuous variables were compared using Student's *t*-test. In case of nonnormal distribution, nonparametric Mann-Whitney *U* test was used for comparison between groups. The difference in the distribution of categorical variables was tested using Chi-square test. Multivariate logistic regression model was employed to identify the independent risk factors associated with CIN. The receiver operating characteristic (ROC) analysis was performed to evaluate the predictive value of SF for CIN and determine the best cutoff value of SF. *P* values < 0.05 were considered statistically significant.

## 3. Results

### 3.1. Baseline Clinical Characteristics

The study population consisted of 548 ACS patients. The mean age of them was 68.6 ± 4.7 years, and 399 (72.8%) were male. Overall, 96 patients (17.5%) developed CIN after PCI procedure. The baseline clinical characteristics of the study population were summarized in Table 1. Patients in the CIN group were significantly older than those in the non-CIN group (68.45 ± 10.32 years versus 61.75 ± 12.30 years, resp.; *P* < 0.001) and had a significantly lower LVEF (*P* < 0.001) compared with patients in the non-CIN group. Statistically significant differences were also noted in history of hypertension or diabetes, type of ACS, and number of diseased vessels (*P* < 0.05) between the two groups. There were no significant differences between the groups regarding gender, body mass index, smoking, systolic blood pressure, dose of contrast medium, hydration therapy, and in-hospital medications (*P* > 0.05).

### 3.2. Baseline Laboratory Data

The biochemical and hematologic parameters were shown in Table 2. SF levels at baseline were significantly higher in patients who developed CIN compared to those who did not (257.05 ± 93.98 versus 211.67 ± 106.65; *P* < 0.001). In addition to having elevated SF, patients in CIN group had significantly higher baseline SCr, hs-CRP, cystatin C, and uric acid levels than those in non-CIN group (*P* < 0.001).

### 3.3. Serum Ferritin for Predicting CIN

To investigate the association between the SF and CIN, multivariate logistic regression analysis was conducted (Table 3). After adjusting for age, history of hypertension and/or diabetes, LVEF < 40%, eGFR < 60 mL/min/1.73 m^2^, levels of uric acid, cystatin C, hs-CRP, clinical diagnosis of acute myocardial infarction (AMI), and 3-vessel disease, SF was still significantly associated with an increased risk of CIN (OR, 1.008; 95% CI, 1.003–1.013; *P* = 0.002). Additionally, hs-CRP (OR, 2.750; 95% CI, 2.028–3.729; *P* < 0.001), renal dysfunction (OR, 9.582; 95% CI, 3.312–27.725; *P* < 0.001), diabetes mellitus (OR, 3.089; 95% CI, 1.101–8.668; *P* = 0.032), ST-elevation myocardial infarction (STEMI) (OR, 3.839; 95% CI, 1.342–10.985; *P* = 0.012), and cystatin C (OR, 1.099; 95% CI, 1.060–1.140; *P* < 0.001) were also independent risk factors of CIN.

### 3.4. Specificity and Sensitivity of Serum Ferritin on Predicting CIN

**Figure 1:**
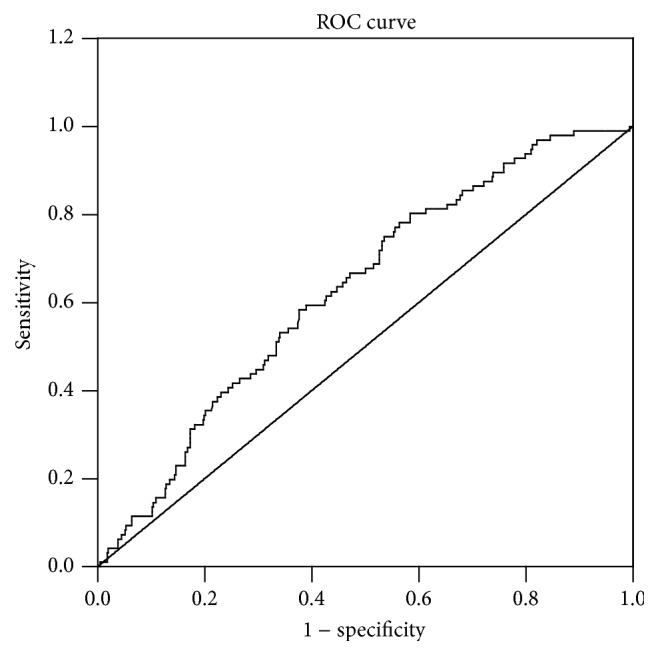
Schematic of the ROC curve for CIN prediction by SF. The area under the ROC curve for predicting CIN of SF was 0.712 with sensitivity of 80.2% and specificity of 41.4%.

The ROC curve analysis (Figure 1) showed that the area under the curve for predicting CIN of SF was 0.629 (95% CI, 0.572–0.686; *P* < 0.001). The optimum cutoff point of SF was 180.9, with sensitivity of 80.2% and specificity of 41.4%.

## 4. Discussion

In the present study, we evaluated the predictive value of SF for the risk of CIN in ACS patients undergoing PCI. Our data suggested that high baseline SF level is significantly associated with an increased risk of CIN.

We also noted that CIN was quite a common complication after PCI for patients with ACS. The incidence of CIN in this study reached up to 17.5% despite performing hydration as a preventive measure in most of the patients. This is consistent with previous reports [3, 15, 16]. The high risk of CIN in patients with ACS should be seriously considered in clinical practice because the onset of CIN would lead to not only extended hospital stays but also irreversible renal functional decline [5]. In addition to ACS, many conditions and disorders predispose to CIN as well, such as preexisting chronic kidney disease, diabetes mellitus, congestive heart failure, advanced age, use of high-contrast media volume, and reduced intravascular volume [17]. In high-risk patients, the incidence of CIN using high osmolar agents was nearly twofold higher than that with low osmolar agents [18]. However, no difference was detected between low osmolar agents and isoosmolar agents applied [19]. Thus, we did not restrict selection to low osmolar or isoosmolar contrast agents in the study.

The precise pathophysiologic mechanisms of CIN have not been fully elucidated yet. Possible mechanisms include renal vasoconstriction, decrease of renal blood flow, formation of reactive oxygen species (ROS), medullary hypoxia, and direct cytotoxicity [20, 21]. Contrast media induce intense vasoconstriction mediated by adenosine, endothelin, calcium influx, and impaired nitric oxide production after a transient phase of vasodilation. These hemodynamic changes lead to sustained renal ischemia particularly in the renal medulla. Medullary hypoxia causes the formation of ROS that may exert direct tubular and vascular endothelial injury and intensify renal parenchymal hypoxia by virtue of endothelial dysfunction [22]. Moreover, contrast media also possess direct cytotoxicity on endothelial and tubular cells, resulting in apoptosis and necrosis [23].

In recent years, studies have demonstrated that inflammatory reaction plays an important role in the pathogenesis of CIN [5]. Several inflammatory cytokines including hs-CRP and interleukin-18 have been demonstrated as independent predictors of CIN after PCI [6–8]. SF is also regarded as a kind of acute-phase protein in some inflammatory diseases, in which significant changes in SF levels have been found [24]. Previous studies have found that SF is closely related to acute renal failure, rapid renal progression in CKD patients, and pathologic changes of diabetic nephropathy [9–11, 25, 26]. However, no relevant report was available regarding whether SF is associated with CIN. Here, we firstly reported that baseline SF level was independently correlated with risk of CIN in ACS patients undergoing PCI. With respect to the mechanisms underlying this association, ROS may play a key role. It has been well documented that production of ROS is central to the progression of many inflammatory diseases [27]. Previous studies showed that SF elicits macrophage accumulation and increases ROS formation, and ROS could exert direct tubular endothelial injury and further exacerbate renal parenchymal hypoxia by means of endothelial dysfunction and dysregulation of tubular transport [22, 28]. Therefore, we propose a hypothesis that SF may be involved in the development of CIN through increasing ROS formation. Further studies are needed to prove this hypothesis.

Moreover, the multivariate logistic regression analysis in this study revealed that hs-CRP, renal dysfunction, diabetes mellitus, STEMI, and cystatin C were independent risk factors of CIN as well. These findings are consistent with earlier reports [1, 4, 7, 29, 30].

There were several limitations to the present study. First, this was an observational study conducted at a single center with a relatively small sample size. A larger study population could make the results more reliable. Second, SF was tested only once at admission. Dynamic observations on the changes of SF and SCr were not conducted.

## 5. Conclusions

In conclusion, our study demonstrated that a higher SF level was significantly associated with an increased risk of CIN after PCI. This in turn suggests that SF may be a useful predictor for CIN after PCI in patients with ACS.

## Figures and Tables

**Table 1:** Baseline clinical characteristics between patients with CIN and those without CIN.

Variables	CIN (*n* = 96)	Non-CIN (*n* = 452)	*P* value
Age (years)	68.45 ± 10.32	61.75 ± 12.30	<0.001
Male, *n* (%)	64 (66.7)	335 (74.1)	0.136
BMI (kg/m^2^)	24.83 ± 6.00	24.13 ± 4.31	0.279
Systolic BP (mmHg)	127.69 ± 20.67	127.82 ± 20.20	0.955
Type of ACS, *n* (%)			<0.001
STEMI	19 (19.8)	32 (7.1)	
NSTEMI	36 (37.5)	121 (26.8)	
UA	41 (42.7)	299 (66.2)	
Smoking, *n* (%)	35 (36.5)	198 (43.8)	0.186
Hypertension, *n* (%)	71 (74.0)	271 (60.0)	0.01
Diabetes mellitus, *n* (%)	57 (59.4)	117 (25.9)	<0.001
LVEF (%)	45.06 ± 9.97	50.30 ± 13.74	<0.001
Number of diseased vessels, *n* (%)			0.034
1	23 (24.0)	150 (33.2)	
2	29 (30.2)	156 (34.5)	
3	44 (45.8)	146 (32.3)	
Contrast dose (mL)	159.9 ± 24.2	156.1 ± 22.1	0.133
Hydration, *n* (%)	62 (64.6)	302 (66.8)	0.674
Medication, *n* (%)			
Aspirin	89 (92.7)	420 (92.9)	0.942
*β*-Blocker	70 (72.9)	335 (74.1)	0.808
Statin	90 (93.8)	415 (91.8)	0.522
ACEI/ARB	69 (71.9)	333 (73.7)	0.717
CCB	24 (25.0)	126 (27.9)	0.566

Data are mean ± standard deviation or number (%). BMI, body mass index; BP, blood pressure; STEMI, ST-elevation myocardial infarction; NSTEMI, non-ST-elevation myocardial infarction; UA, unstable angina; LVEF, left ventricular ejection fraction; ACEI, angiotensin-converting enzyme inhibitor; ARB, angiotensin receptor blocker; CCB, calcium channel blocker.

**Table 2:** Baseline laboratory data between patients with CIN and those without CIN.

Variables	CIN (*n* = 96)	Non-CIN (*n* = 452)	*P* value
SF (*μ*g/L)	257.05 ± 93.98	211.67 ± 106.65	<0.001
hs-CRP (mg/L)	15.45 ± 2.60	10.25 ± 2.37	<0.001
Hemoglobin (g/L)	116.85 ± 20.30	120.14 ± 25.79	0.173
Cystatin-C (mg/L)	1.34 ± 0.18	0.84 ± 0.20	<0.001
LDL-C (mmol/L)	3.22 ± 0.86	3.29 ± 1.00	0.540
Uric acid (mmol/L)	462.72 ± 74.66	410.49 ± 70.75	<0.001
Creatinine (*μ*mol/L)	113.21 ± 20.93	85.58 ± 18.57	<0.001
eGFR (mL/min/1.73 m^2^)	57.70 ± 9.89	77.06 ± 16.30	<0.001

Data are mean ± standard deviation or number (%). SF, serum ferritin; Hs-CRP, high-sensitivity C-reactive protein; LDL-C, low-density lipoprotein cholesterol; eGFR, estimated glomerular filtration rate.

**Table 3:** Univariate and multivariate logistic regression analysis of CIN risk factors.

Variables	Univariate analysis			Multivariate analysis		
	OR	95% CI	*P*	OR	95% CI	*P*
Age	1.048	1.028–1.069	<0.001	1.046	0.997–1.097	0.068
Hypertension	1.897	1.158–3.106	0.011	1.975	0.671–5.807	0.216
Diabetes mellitus	4.185	2.646–6.619	<0.001	3.089	1.101–8.668	0.032
LVEF < 40%	1.524	0.934–2.484	0.091	—	—	—
Uric acid	1.010	1.007–1.013	<0.001	1.004	0.997–1.012	0.227
SF	1.004	1.002–1.006	<0.001	1.008	1.003–1.013	0.002
eGFR < 60 mL/min/1.73 m^2^	8.045	4.970–13.022	<0.001	9.582	3.312–27.725	<0.001
Cystatin-C	1.088	1.069–1.107	<0.001	1.099	1.060–1.140	<0.001
hs-CRP	2.334	1.985–2.743	<0.001	2.750	2.028–3.729	<0.001
3-Vessel disease	1.792	1.145–2.802	0.011	0.880	0.302–2.566	0.880
STEMI	2.622	1.673–4.107	<0.001	3.839	1.342–10.985	0.012

LVEF, left ventricular ejection fraction; SF, serum ferritin; eGFR, estimated glomerular filtration rate; Hs-CRP, high-sensitivity C-reactive protein; STEMI, ST-elevation myocardial infarction.
